# Supplementary material for: Recurrent horizontal transfer identifies mitochondrial positive selection in a transmissible cancer
Source: Nat Commun. 2020 Jun 16;11:3059. doi: 10.1038/s41467-020-16765-w (PMC7297733; doi:10.1038/s41467-020-16765-w)
Supplement: Supplementary file 1 — Supplementary Information [file 41467_2020_16765_MOESM1_ESM.pdf]

Supplementary Information for

Recurrent horizontal transfer identifies mitochondrial positive selection in a transmissible cancer

Strakova et al.

This file contains:

Supplementary Figures 1-8
Supplementary References

Other Supplementary Materials for this manuscript include the following:

Supplementary Data 1-6

Supplementary Figure 1

Nuclear tree

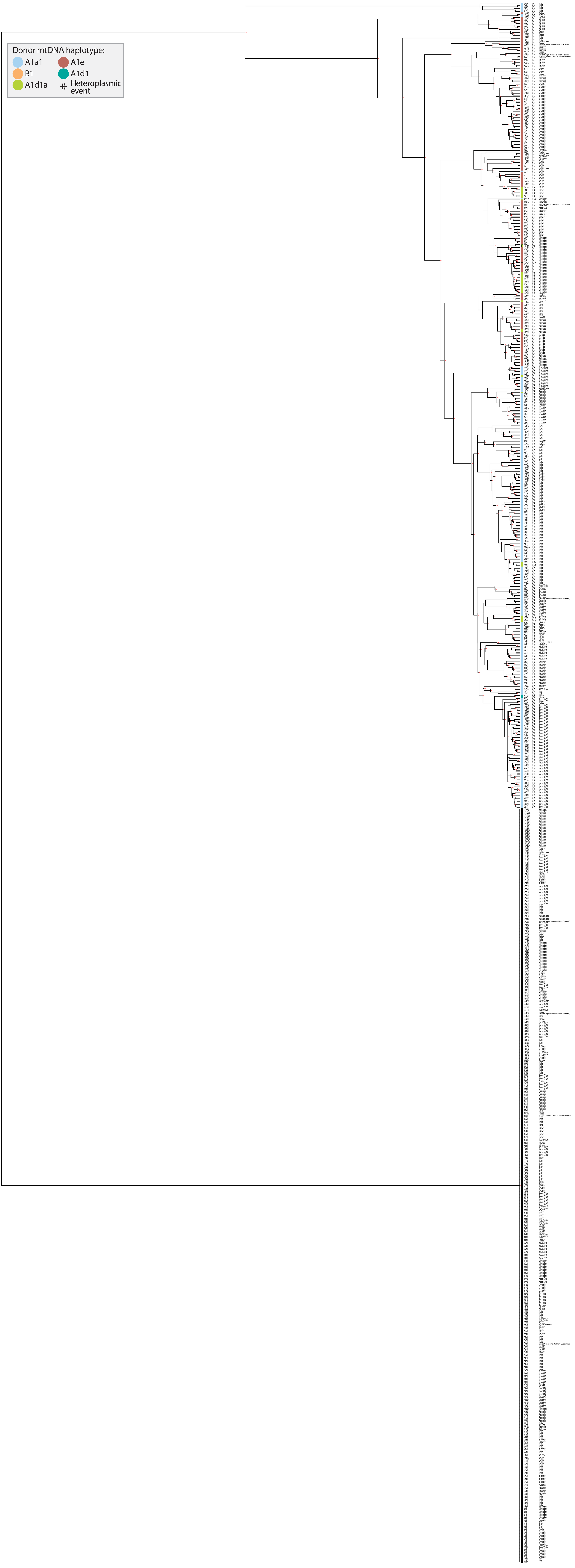

Supplementary Figure 1: Nuclear phylogenetic tree.
Maximum likelihood phylogenetic tree constructed using 148,030 nuclear CTVT somatic mutations genotyped across 539 CTVTs (coloured) and 494 CTVT host dogs (black) [1]. Samples are labelled with identifier, country and, in the case of tumours, horizontal transfer (HT) group coloured by donor haplotype (Supplementary Data 1). Heteroplasmic horizontal transfer events are indicated by asterisks (*) (Table 1). Branch lengths are roughly proportional to the number of somatic mutations per branch and bootstrap support for each node is indicated in red.

Supplementary Figure 2

mtDNA tree

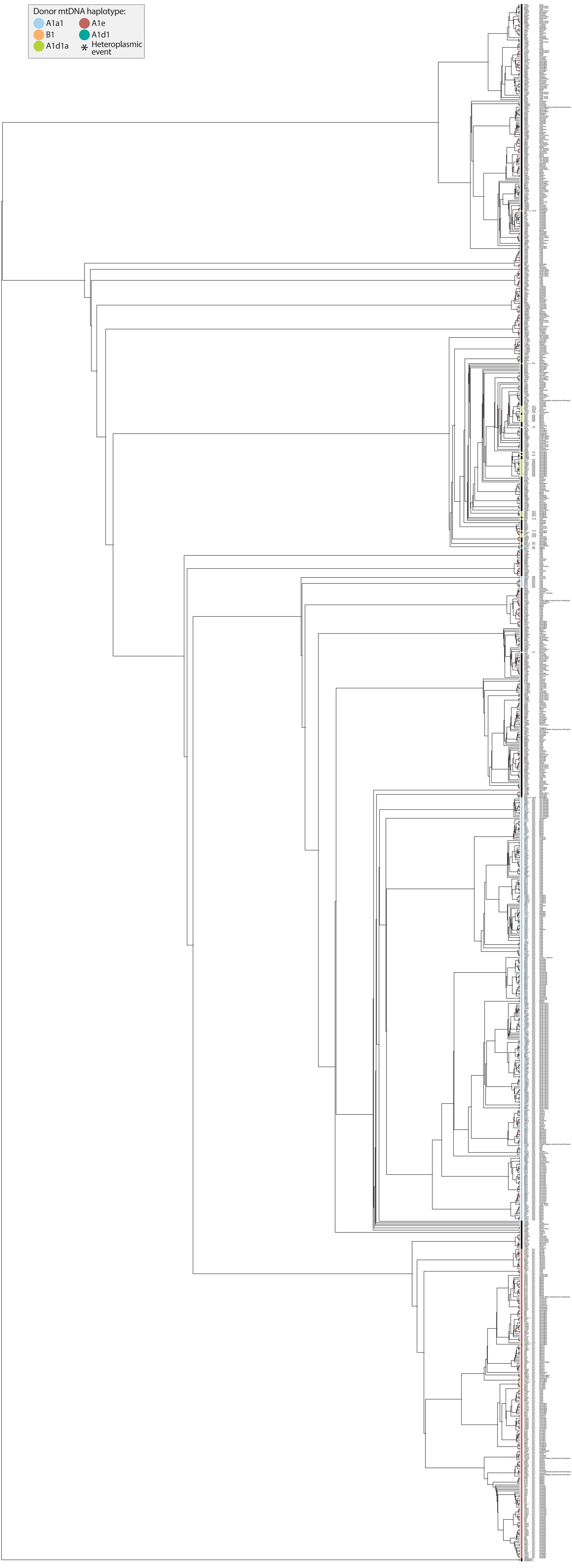

Supplementary Figure 2: mtDNA phylogenetic tree.
Maximum likelihood phylogenetic tree constructed with mtDNAs from the same set of 539 CTVTs and 494 host dogs shown in Supplementary Figure 1. Distinct haplotypes present in heteroplasmic samples (see Table 1, Supplementary Data 1 and 2) are placed individually on the tree and marked by asterisks (*). Samples are labelled with identifier and country and, in the case of tumours, horizontal transfer (HT) group coloured by donor haplotype (Supplementary Data 1). Tree is presented as a cladogram and branch lengths are not informative.

Supplementary Figure 3

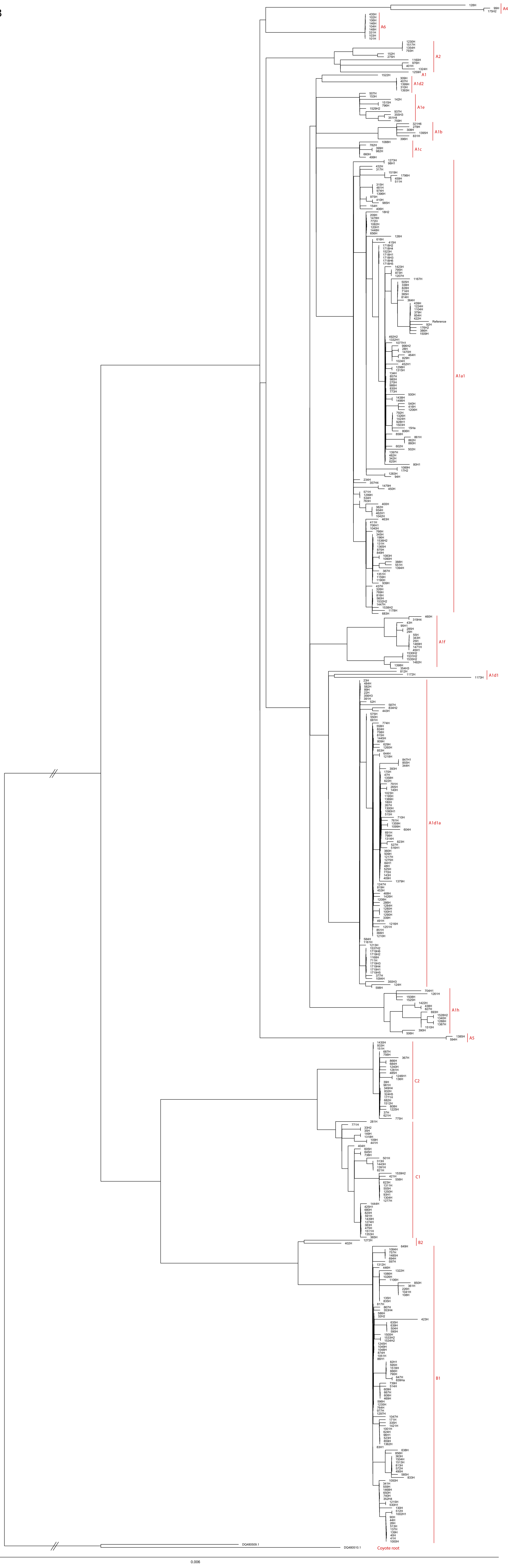

Supplementary Figure 3: Dog haplotypes mtDNA phylogenetic tree.

Maximum likelihood phylogenetic tree constructed with mtDNAs from the same set of 494 CTVT host dogs shown in Supplementary Figures 1 and 2. Samples are labelled with identifier (Supplementary Data 1). Haplotypes are indicated using red lines (haplotype naming based on [2]). Branch lengths are proportional to the number of mitochondrial variants per branch, with scale expressed in base substitutions per site. Branches leading to the coyote outgroup have been shortened in order to aid visualisation (indicated by double forward slash, //).

Supplementary Figure 4

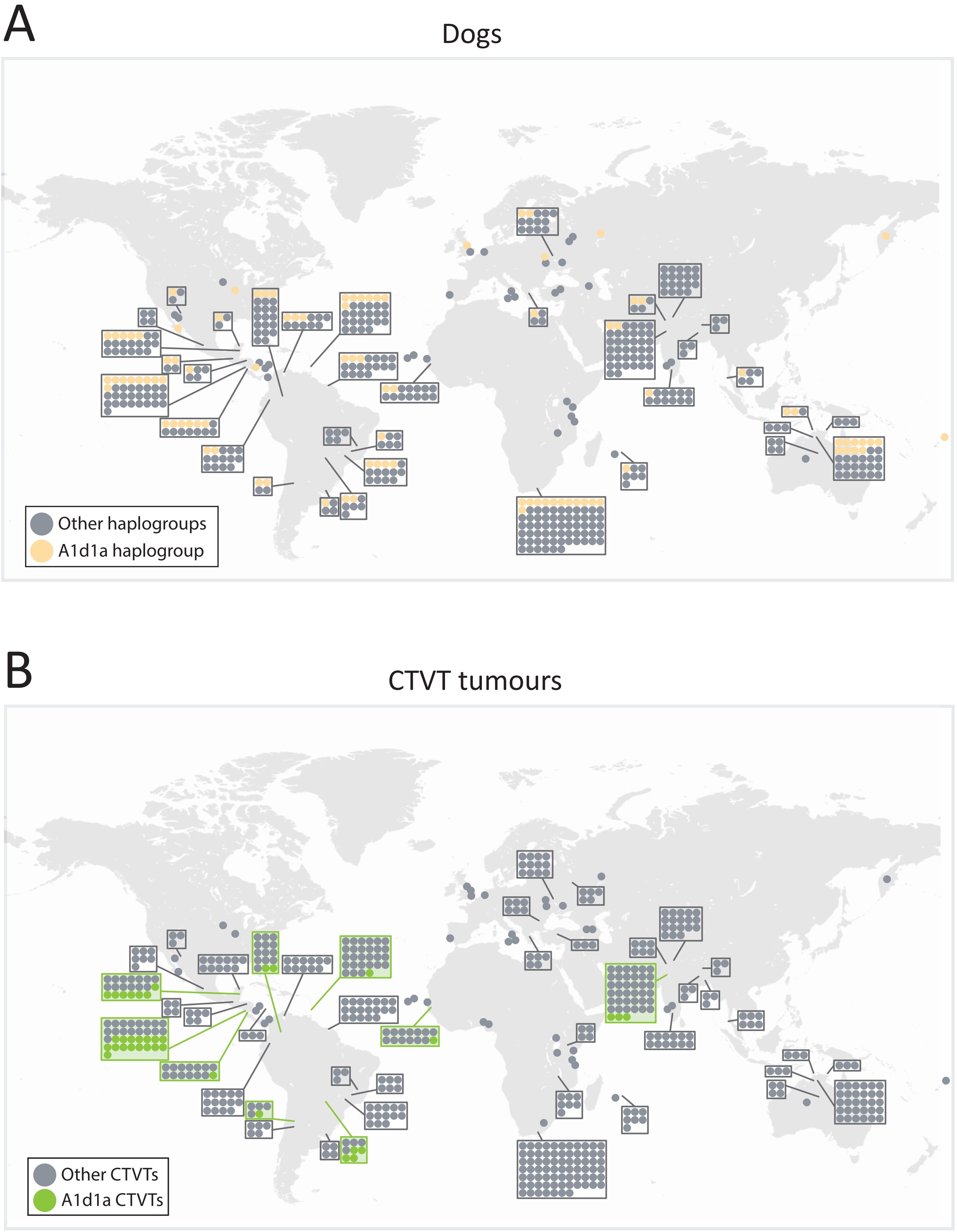

Supplementary Figure 4: A1d1a CTVT and dog geographical distribution.
Geographical locations of 539 CTVT tumours and 495 CTVT host dogs included in this study. Each dot represents the location of (A) a dog or (B) a CTVT tumour. Dogs and CTVTs with A1d1a haplotypes are indicated in colour (see Supplementary Data 1 and 2).

Supplementary Figure 5

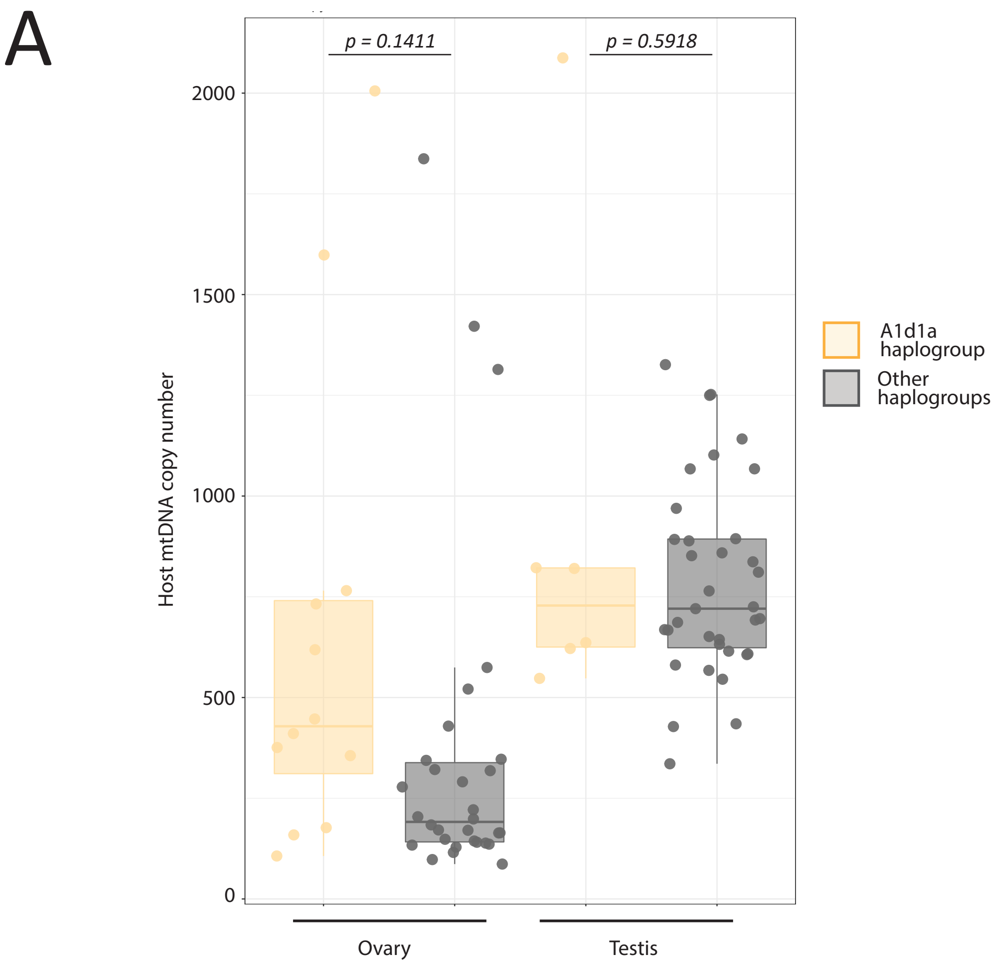

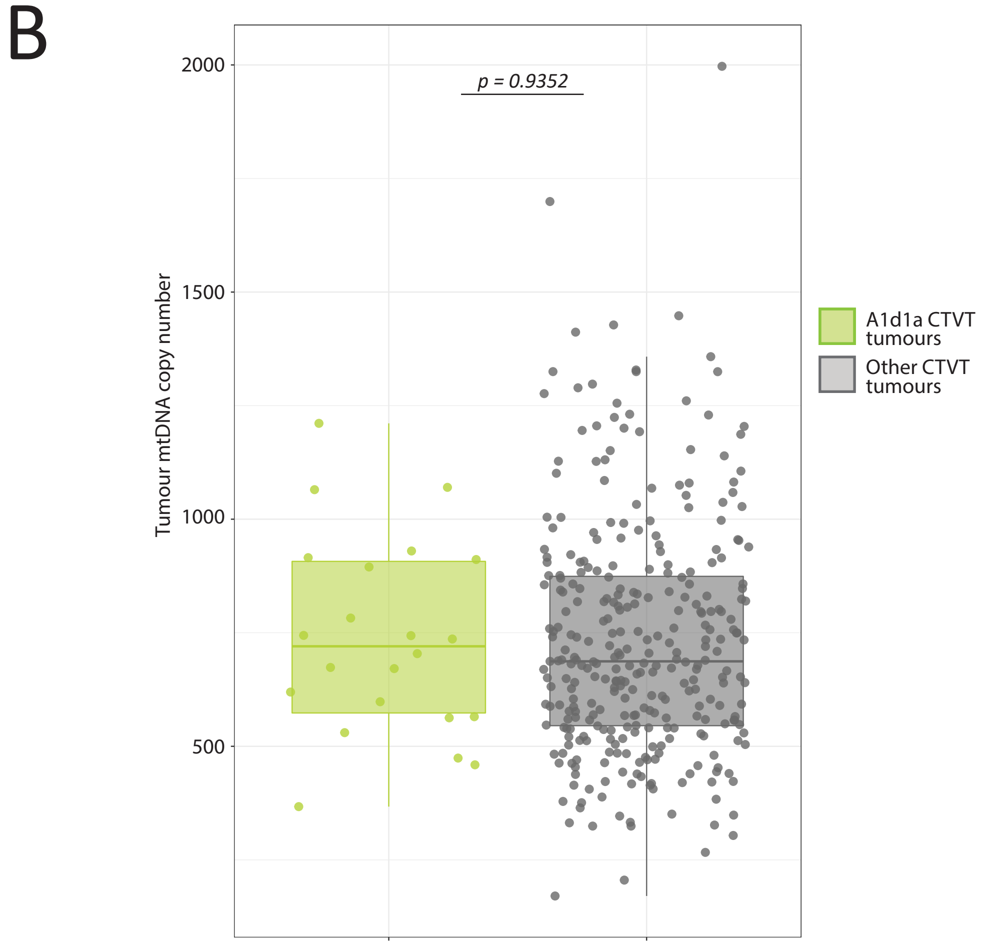

Supplementary Figure 5: A1d1a mtDNA copy number.

(A) MtDNA copy number of the A1d1a haplotype and other haplotypes in normal canine ovarian (n=42) and testicular (n=41) tissue. Each individual is represented by a dot. Boxes represent the first and third quartiles (inter-quartile range, IQR). Middle lines within each box represent the median. Error bars indicate values within 1.5 times the IQR from the first and third quartiles. P-values were calculated using an unpaired two-tailed Student's t-test. (B) MtDNA copy number in A1d1a CTVT tumours (n=22) and in tumours with other mtDNA haplotypes (n=315). Each tumour is represented by a dot. Data and p-values are calculated and displayed as in (A).

Supplementary Figure 6

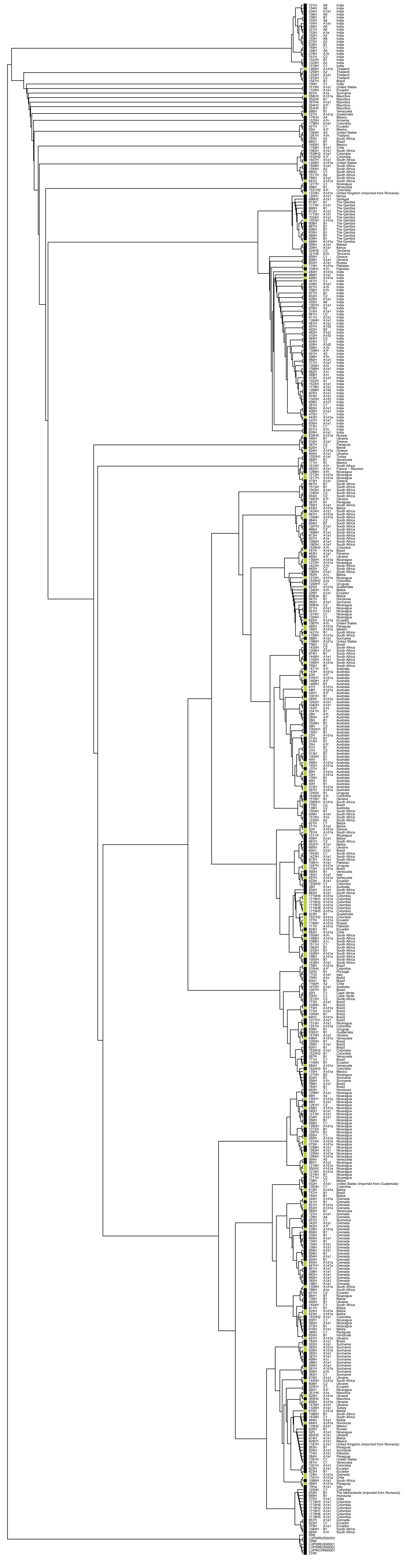

Supplementary Figure 6: Dog nuclear genome phylogenetic tree.
Neighbour-joining cladogram constructed using 136,880 canine germline variants genotyped across 495 CTVT host dogs and seven wolves. Each sample is labelled with an identifier, mtDNA haplotype and country (Supplementary Data 1). A1d1a dogs are indicated in green. Branch-lengths are not informative.

Supplementary Figure 7

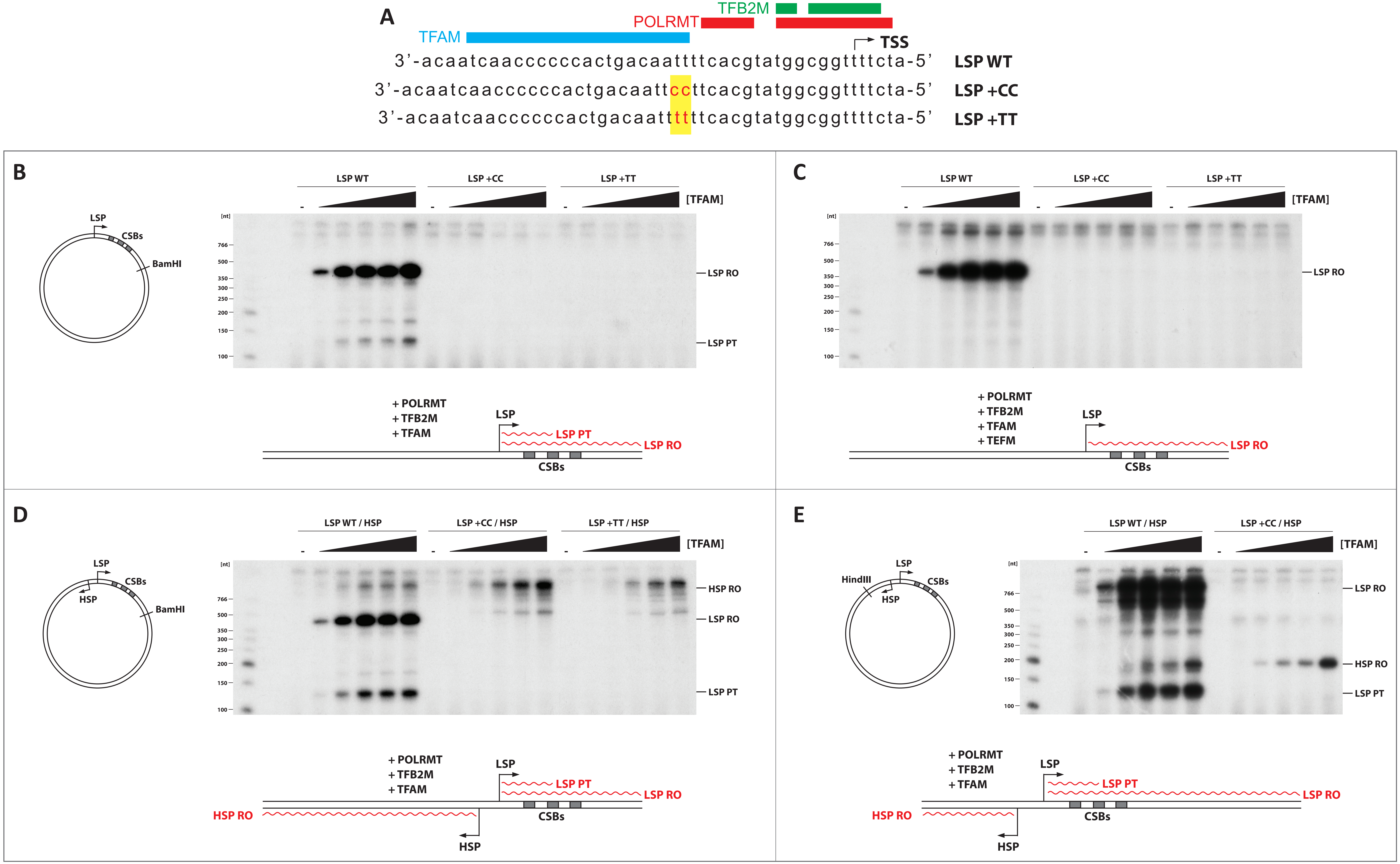

Supplementary Figure 7: MtDNA transcription assays.

MtDNA *in vitro* transcription assays using a human template with mutations introduced at an equivalent region to canine 16660insCC. (A) Sequence of human LSP, indicating transcription start site (TSS) and regions contacted by TFAM, POLRMT and TF2BM proteins (coloured boxes). Yellow box indicates site of inserted residues to create LSP +CC and LSP +TT constructs, whose transcriptional activity was compared with that of the wild type (WT) construct. (B) Transcription reactions using LSP templates (WT, +CC and +TT). Reactions contain 90 fmol BamHI-linearised template DNA, 500 fmol POLRMT, 500 fmol TFB2M, and varying amounts of TFAM (0, 0.1, 1, 2.5, 5 and 10 pmol). 'LSP RO' indicates the runoff transcript, and 'LSP PT' indicates pretermination of transcription at CSB2. Transcription reactions in (B)-(D) were repeated at least twice independently. (C) Transcription reactions as in (B) with the addition of 1 pmol TEFM. (D) Transcription reactions as in (B) using a template that contains the entire promoter region, spanning both LSP and HSP. (E) Transcription reactions as in (D) but using templates linearised using HindIII to generate a shorter runoff product from transcription initiated at HSP.

Supplementary Figure 8

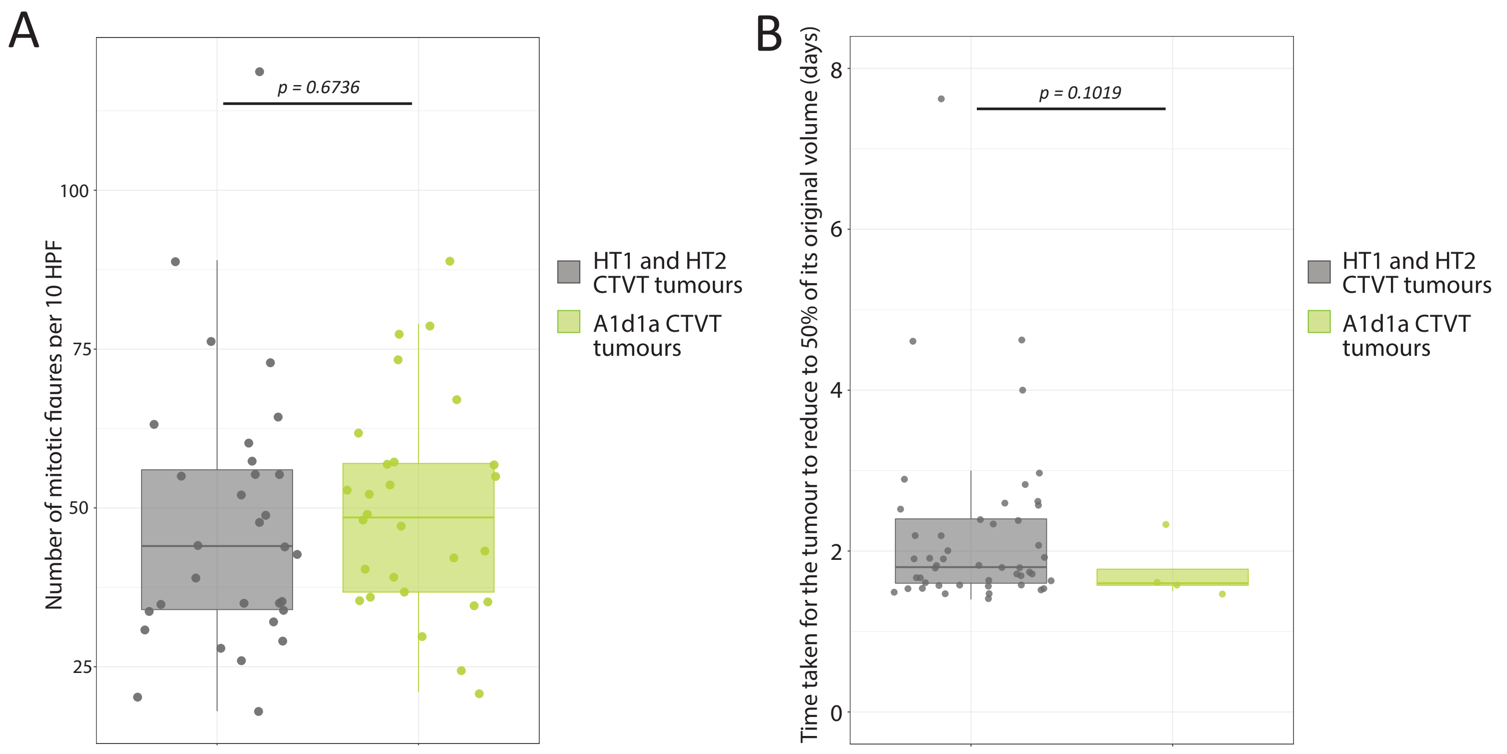

Supplementary Figure 8: Analysis of possible A1d1a adaptive phenotypes.
(A) Number of mitotic figures per 10 high-power fields (HPFs) in A1d1a CTVTs (n=28) and CTVT_HT1 and CTVT_HT2 CTVTs (n=31) (Materials and Methods). The 28 A1d1a CTVTs belong to 8 horizontal transfer groups (HT3, 2 tumours; HT8, 6 tumours; HT9, 13 tumours; HT10, 1 tumour; HT13, 1 tumour; HT14, 1 tumour; HT16, 1 tumour; HT17, 3 tumours). Each dot represents a single tumour. Boxes represent the first and third quartiles (inter-quartile range, IQR). Middle lines within each box represent the median. Error bars indicate values within 1.5 times the IQR from the first and third quartiles. P-value was calculated using an unpaired two-tailed Student's t-test. (B) CTVT response to vincristine chemotherapy treatment. Four A1d1a CTVTs and 48 CTVT_HT1 and CTVT_HT2 CTVTs were treated with intravenous vincristine sulfate (0.02-0.025mg/kg or 0.5mg/m2) and the time taken (days) for the tumour to reduce to 50% of its original volume was measured. Each dot represents a single tumour. Data are displayed and p-values calculated as in (A). A single tumour belonging to the CTVT_HT1 and CTVT_HT2 group that did not reach 50% of its original volume within the study period was excluded from this analysis.

Supplementary References

1. Baez-Ortega, A. *et al.* Somatic evolution and global expansion of an ancient transmissible cancer lineage. *Science* **365**, eaau9923 (2019).

2. Fregel, R. *et al.* Mitochondrial DNA haplogroup phylogeny of the dog: Proposal for a cladistic nomenclature. *Mitochondrion* **22**, 75-84 (2015).

3. Strakova, A. *et al.* Mitochondrial genetic diversity, selection and recombination in a canine transmissible cancer. *Elife* **5**, 10.7554/eLife.14552 (2016).

4. McLaren, W. *et al.* Deriving the consequences of genomic variants with the Ensembl API and SNP Effect Predictor. *Bioinformatics* **26**, 2069-70 (2010).
