# Supplementary material for: Recurrent horizontal transfer identifies mitochondrial positive selection in a transmissible cancer
Source: Nat Commun. 2020 Jun 16;11:3059. doi: 10.1038/s41467-020-16765-w (PMC7297733; doi:10.1038/s41467-020-16765-w)
Supplement: Supplementary file 3 — Description of Additional Supplementary Files [file 41467_2020_16765_MOESM3_ESM.pdf]

Description of Additional Supplementary Files

Recurrent horizontal transfer identifies mitochondrial positive selection in a transmissible cancer, Strakova et al.

File Name: Supplementary Data 1
Description: Summary of information available for 539 CTVT tumours and 495 dogs analysed in this study. Most of the samples (478) are matched tumour-host dog pairs. 491/495 dog germline samples are CTVT hosts and 4/495 are CTVT-unaffected dogs; however, 14/491 CTVT hosts do not have matched tumours included in the study due to failure of tumours to pass quality filters. Samples from two dogs were sequenced multiple times and were included as technical replicates. Table includes available data on location, year of collection, CTVT horizontal transfer group, dog mtDNA haplotype, breed, age and sex.

File Name: Supplementary Data 2
Description: Single-nucleotide variant lists for (A) 539 CTVT tumours and (B) 495 CTVT host dogs analysed in this study. (C) Indel genotypes at position 16660 for tumour and host cohorts. Distinct haplotypes present in CTVT tumour heteroplasmic samples (see Table 1, Supplementary Data 1), and technical replicates for two CTVT host dogs that were sequenced multiple times (see Supplementary Data 1) are listed individually. CTVT horizontal transfer group and host haplotype group is listed for each sample.

File Name: Supplementary Data 3
Description: Summary of canine haplotype frequency in CTVT host dog population (n=495 dog mitochondrial genomes) and the frequency of horizontal transfer in each group.

File Name: Supplementary Data 4
Description: Mean and range of somatic mutations observed within each horizontal transfer (HT) group. Somatic mutations are defined as variants arising after each horizontal transfer event that are polymorphic within an HT group. In addition, the number of 'potential-somatic' mutations of unknown somatic or germline status is listed; these are variants that are common to all tumours within the HT group and which are not present on the most closely related normal canine haplotype. These may represent rare germline variants that have not been captured by our normal panel or somatic mutations that occurred prior to tumour divergence within each HT group (Methods). Number of potential somatic variants in HT1 is influenced by mtDNA recombination that occurred in one phylogenetic branch of HT1 tumours [3]. Using a CTVT mtDNA mutation rate of 0.0201 mutations per year (Methods) [1,3], the estimated time since each HT event is listed.

File Name: Supplementary Data 5
Description: (A) Genetic variation unique to A1d1a (or shared with A1d1, as indicated). (B) Genetic variation shared between A1d1a and other dog haplotypes. Annotation in parts (A) and (B) was performed using Variant Effect Predictor [4].

File Name: Supplementary Data 6
Description: (A) List of CTVTs analysed using RNA sequencing and their respective horizontal transfer (HT) events and groups (see Fig. 3a), sample batches and tumour purity estimates. The CTVT_HT1/HT2+insCC/insC/Rec haplotypes are as follows: 335Ta, CTVT_HT2 carrying 16660insCC somatic mutation; 341Ta, CTVT_HT2 carrying 16660insC somatic mutation; 559Ta, recombinant CTVT_HT1/A1d1a carrying 16660insCC; 560Ta, CTVT_HT1 carrying 16660inCC somatic mutation. Tumour purity estimates were unavailable for three tumours. (B) MtDNA transcript abundance per gene. Table includes information on gene name, gene symbol, chromosome (Chrom), estimated

relative transcript abundance per sample (transcripts per million), and log2 fold changes and Wald test *p* values for differences between CTVT_A1d1a and CTVT_HT1/CTVT_HT2 gene expression (in normalised gene counts, accounting for sample batches; Methods). (C) Nuclear mitochondrial transcript abundance per gene. Information is displayed as in (B).
